# Neutrophils in Tuberculosis: Heterogeneity Shapes the Way?

**DOI:** 10.1155/2017/8619307

**Published:** 2017-05-24

**Authors:** Irina V. Lyadova

**Affiliations:** Immunology Department, Central Tuberculosis Research Institute, Yauza Alley 2, Moscow 107564, Russia

## Abstract

Infection with *M. tuberculosis* remains one of the most common infections in the world. The outcome of the infection depends on host ability to mount effective protection and balance inflammatory responses. Neutrophils are innate immune cells implicated in both processes. Accordingly, during *M. tuberculosis* infection, they play a dual role. Particularly, they contribute to the generation of effector T cells, participate in the formation of granuloma, and are directly involved in tissue necrosis, destruction, and infection dissemination. Neutrophils have a high bactericidal potential. However, data on their ability to eliminate *M. tuberculosis* are controversial, and the results of neutrophil depletion experiments are not uniform. Thus, the overall roles of neutrophils during *M. tuberculosis* infection and factors that determine these roles are not fully understood. This review analyzes data on neutrophil defensive and pathological functions during tuberculosis and considers hypotheses explaining the dualism of neutrophils during *M. tuberculosis* infection and tuberculosis disease.

## 1. Introduction

Inflammatory response is a part of host biological response to infection. As such, it is aimed to protect host against invading organisms and sustain host homeostasis. However, the response can also be detrimental and contribute to pathology. The dual role of inflammation is especially apparent during chronic infections, due to permanent immune stimulation by pathogen-derived signals.

Tuberculosis (TB) is an infectious disease, in which the extent and the quality of host inflammatory reactions play an exceptionally significant role in both protection and pathology. At the initial stage of *M. tuberculosis* (*Mtb*) infection, proinflammatory cytokines and chemokines released by diverse cells induce immune cell migration to the infectious site, start granuloma formation, and initiate host protective responses. Cellular populations implicated in the responses involve alveolar macrophages, dendritic cells (DC), neutrophils, NK cells, epithelial cells, and other cells. Further protection against *Mtb* largely relies on T lymphocytes, particularly, Th1 effector cells [1, 2]. Th1 lymphocytes operate primarily by secreting a wide range of proinflammatory factors able to activate macrophages for *Mtb* killing (e.g., IFN-*γ*, TNF-*α*), recruit new immune cells at the infectious site (e.g., CCL2/MCP-1, CCL3/MIP-1*α*, CCL4/MIP-1*β*, CCL5/RANTES, and GM-CSF), and mediate granuloma formation [3, 4]. The generation of Th1 lymphocytes is driven by pathogen-specific antigens and governed by several cytokines secreted by innate immune cells. Deficiency in CD4 cells or cytokines involved in Th1 generation and/or function results in severe experimental *Mtb* infection in mice and increased risk of mycobacterial infections in humans [5–9]. Thus, TB is often regarded as a disease that develops due to immune deficiency.

On the other hand, since Koch's studies, TB has been considered an immunopathological disease, developed due to immune hyperreactivity. Immunological reactions associated with TB pathology involve uncontrolled secretion of proinflammatory cytokines and chemokines, extensive neutrophilic infiltration, and exacerbated T cell responses, including those of Th1 cells [10–14]. Thus, the same immune cells that are needed for the protection are implicated in TB pathology.

Among immune cell populations, playing a dual role during TB, probably least understood are neutrophils. Up to now, the information on the role for neutrophils in TB protection and pathology is highly contradictory, with some data implicating neutrophils in the TB control and others associating them with TB pathology.

In this review, we analyze data on neutrophil defensive and pathological functions during TB and consider hypotheses explaining the dualism of these innate immune cells during *Mtb* infection and TB disease.

## 2. Neutrophil Bactericidal Activity

### 2.1. General Properties

Neutrophils are short-living and at the same time the most abundant leukocytes in the blood. Following infection, neutrophils arrive first at the inflammatory/infectious site [15]. The process is multistep and requires interactions between multiple neutrophil receptors (such as G-protein-coupled receptors (GPCR), Toll-like receptors, nucleotide-binding oligomerization domain-like receptors, C-type lectins, and cytokine and chemokine receptors) and microbial products, endothelial cell receptors, molecules released by dying cells, and inflammatory cytokines and chemokines (reviewed in details in [16–18]).

Upon their arrival at the infectious site, neutrophils phagocytose bacteria recognizing them either directly or through Fc*γ* and complement receptors [19]. Phagocytosis and subsequent pathogen killing require neutrophil activation that is a two-step process and includes initial neutrophil priming. The latter depends on neutrophil exposure to cytokines (e.g., TNF-*α*, IL-1*β*), pathogen-associated molecular patterns (PAMPs), chemokines, and growth factors (e.g., CXCL2/MIP-2*α*, LTB4, and GM-CSF) or cell interaction with activated endothelial surfaces [18, 20].

Killing of engulfed bacteria is mediated through the degranulation, the generation of reactive oxygen intermediates (ROI), and the formation of neutrophil extracellular traps (NETs). Following the degranulation, granule-associated bactericidal proteins and peptides are discharged into the microbe-containing phagocytic vacuole. Neutrophil bactericidal molecules are numerous and include neutral proteinases cathepsin G, elastase, and proteinase 3; bactericidal/permeability-increasing protein (BPI); defensins (e.g., human neutrophil proteins 1–3, HNP-1–3); cathelicidin LL-37; lactoferrin; and lysozyme [19, 21, 22]. ROI are generated by NADPH-dependent oxidase and superoxide dismutase. Hypochlorous acid and chloramines are generated by metalloperoxidase. Activated neutrophils can also produce nitric oxide (NO), although much less efficient than ROI, and peroxynitrate, a highly reactive product of nitric oxide oxidation [16]. Besides discharging granule-derived mediators into the phagosomes, neutrophils also release them extracellularly, which helps in killing extracellular bacteria, but also causes tissue damage. Extracellular release largely occurs during the formation of NETs that are composed of a web of DNA, histones, and granule-derived antimicrobial proteins and function to kill or at least restrict the growth of extracellular bacilli [16, 23–25]. Neutrophil degranulation, respiratory burst, and NET formation are stimulated by bacteria products and inflammatory molecules signaling through TLRs, Fc receptors, GPCR, and receptors for TNF-*α*, IFN-*γ*, and IL-18 [18].

Overall, neutrophils have evolved into efficient pathogen-killing machinery. Their essential role in the resistance to various bacterial and fungal infections is confirmed by the development of progressive infections with a wide range of organisms in neutropenia conditions [22].

### 2.2. Bactericidal Activity of Neutrophils during *Mtb* Infection

During *Mtb* infection, neutrophils are among the first cells that migrate to the infectious focus [15]. The ability of neutrophils to phagocyte *Mtb* has been demonstrated in many studies, both in vitro and in vivo [26–29]. Particularly, in vivo, neutrophils accumulated in the lung tissue and in the airspaces of mice challenged with BCG or *Mtb* one-day postchallenge and 1.6% of neutrophils contained mycobacteria [28]. In isolated human lung tissue infected in vitro with various mycobacterial strains, approximately 7% of the infected cells were neutrophils [29].

In contrast to the phagocytosis, data on neutrophil capacity to kill *Mtb* are conflicting. Several in vitro studies reported poor antimycobacterial activity of neutrophils, even when the cells were stimulated with IFN-*γ*, a cytokine known for its ability to activate antimycobacterial properties of macrophages [27, 30]. Other groups showed that neutrophils and neutrophil-derived bactericidal molecules do kill *Mtb* in vitro [26, 31–33]. In the study by Kisich and coauthors [26], mycobacterial killing depended on the activation of neutrophils by TNF-*α*, suggesting that poor mycobactericidal activity of neutrophils observed in other studies could be attributed to an inappropriate cell stimulation. However, in some studies, neutrophils did not alter *Mtb* survival even upon priming with TNF-*α* [34, 35]. In the study by Corleis and coauthors, neutrophils did not kill *Mtb* but were able to kill *M. smegmatis* and mutant *Mtb* H37RvΔRD1 strain, demonstrating that *Mtb* escape from neutrophil-mediated killing depends on the RD1 virulence region [35].

A part of neutrophil bactericidal activity is mediated by NETs. Neutrophils stimulated by *Mtb* in vitro were shown to release NETs containing neutrophil elastase and histones, yet they were unable to kill *Mtb* [36]. Furthermore, it was suggested that NETs may provide a platform for extracellular *Mtb* growth and in this way contribute to the rapid enlargement of the pulmonary lesions [37, 38].

A poor capacity of neutrophils for *Mtb* killing allowed some authors to consider them “Trojan horse” hiding *Mtb* from potentially bactericidal macrophages [27, 39]. On the other side, neutrophils were shown to increase the bactericidal activity of macrophages: in the study by Tan and coauthors, macrophages phagocyted apoptotic neutrophils and utilized their bactericidal peptides to combat intracellular *Mtb* [40]. Data on the ability of neutrophils to kill *Mtb* in vivo are also conflicting. Neutrophils found in the sputum and BAL fluids of patients with active pulmonary TB were shown to contain replicating *Mtb*, which was considered an indication of cell inability to control the pathogen [41]. On the other hand, multiple associative studies have linked neutrophils to the protection against TB disease. Particularly, in TB contacts, the counts of peripheral blood neutrophils inversely correlated with the risk of TB development. In the same study, Black African participants (known to have high susceptibility to TB) had lower counts of neutrophils and lower concentrations of circulating HNP1–3 and lipocalin 2 peptides compared to White participants [33]. In another study, low plasma levels of HNP1–3 have been associated with the development of multidrug-resistant TB [42].

While an association between TB protection and neutrophil counts may be indirect (e.g., mediated by neutrophil-dependent activation of other immune cells), an association between the protection and HNP-1–3 levels suggests the direct involvement of neutrophils in TB prevention. In line with this, several experimental studies reported antimycobacterial effects of neutrophils in vivo. In rats, LPS-induced transient neutrophilia reduced pulmonary CFU counts following subsequent animal challenge with *Mtb*, and neutrophils obtained by bronchoalveolar lavage of rats 24 h after the intratracheal introduction of LPS (i.e., in vivo activated neutrophils) were able to kill *Mtb* in vitro [43]. In zebrafish, granuloma-associated neutrophils phagocyted *Mycobacterium marinum* and destroyed them utilizing NADPH oxidase-dependent mechanism [44]. Mincle, the member of C-type lectin superfamily, is expressed by neutrophils and recognizes trehalose dimycolate (TDM), a cord factor of *Mtb*. The recognition leads to neutrophil activation. In *Mincle*^−/−^ mice, the recruitment of neutrophils to the lungs was reduced which was accompanied by increased mycobacterial loads, supporting the involvement of neutrophils in early protection against *Mtb* [45].

To summarize, there is a great controversy as to whether neutrophils are able to kill *Mtb*. The question on why these cells with exceptionally high bactericidal potential are not unequivocally bactericidal against mycobacteria is yet to be answered.

## 3. Neutrophil Interactions with Other Immune Cells

### 3.1. General Properties

Neutrophils secrete a wide range of cytokines, chemokines, and enzymes, such as IL-1*β*, IL-1*α*, TNF-*α*, CXCL1/KC, CXCL8/IL-8, CCL3/MIP-1*α*, CCL4/MIP-1*β*, GM-CSF, and metalloproteinases (MMPs) [46–52]. Factors secreted by neutrophils and molecules expressed on their surface underlie neutrophil cross-talk with other immune cells and induce immune cell activation, recruitment to the infectious site, and immune response generation and regulation.

Among different immune cells affected by neutrophil-derived factors are neutrophils themselves: IL-1*β* and TNF-*α* stimulate neutrophil migration, degranulation, oxidative burst, and secretory activity; CXCL1 and CXCL8 are the main neutrophil-attracting factors; and MMP-8 and elastase hydrolyse extracellular matrix proteins facilitate the process of immune cell migration throughout the extracellular matrix and cleave chemokines increasing their attractant activity (e.g., CXCL5 [53]).

Lacteferrin, *α*-defensins, and chemokines released by neutrophils are attractant for DC, monocytes, and lymphocytes. Binding of neutrophils to DC promotes DC maturation [16]. DC are able to internalize neutrophils acquiring pathogen antigens and cross-presenting them to T lymphocytes [54, 55]. Thus, a role for neutrophils in “concentrating” antigens has been proposed [55].

For T lymphocytes, neutrophils can serve as antigen-presenting cells [56, 57]. Neutrophils were shown to carry antigens from the peripheral sites to the lymph nodes and bone marrow and facilitate the generation of Th1, Th17, and CD8^+^ memory responses [57–59]. NETs released by human neutrophils can directly prime T cells by reducing their activation threshold [60]. Neutrophils can also activate NK cells and support the survival of B lymphocytes, basically through the production of BAFF (reviewed in [16]).

Of particular importance for TB are neutrophil interactions with macrophages (reviewed in details in [61]). Chemokines, granule proteins, and other molecules released by neutrophils (e.g., CCL2, CCL3, CCL19, CCL20, S100A8, and S100A9) recruit monocytes to the site of infection [16]. Macrophages phagocyte apoptotic neutrophils by efferocytosis, which leads to several consequences, that is, removing neutrophils and preventing tissue injury, allowing macrophages to utilize neutrophil granule proteins for antimicrobial defense, and altering cytokine production by macrophages [40, 62, 63]. The latter depends on neutrophil-derived signals and inflammatory milieu. In inflammatory conditions, efferocytosis enhances IL-10 and/or TGF-*β* production stimulating M2 polarization and the resolution of inflammation [62, 64].

Neutrophils can also exert immunoregulatory activity towards other immune cells. Particularly, they can inhibit proliferation and IFN-*γ* production by T lymphocytes, shut down Th17 cells, and limit *γδ*T cell function using IL-10, arginase-I, and ROS-dependent mechanisms [59, 65].

### 3.2. Neutrophils in T Cell Activation and Early Granuloma Formation during TB

At the setting of *Mtb* infection, the interactions of neutrophils with DC and T lymphocytes are well documented. The studies are not numerous, but uniform. In mice infected with *Mtb*, neutrophils are among the first cells to arrive at the infectious site and their peak precedes the peak of the infected DC in the lungs. Neutrophils increased trafficking of DC to the lymph nodes and captured and delivered *Mtb* to DC in a form that made DC more effective initiators of CD4 T cell activation [66]. Following subcutaneous inoculation of BCG, neutrophils phagocyted mycobacteria and carried them to the draining lymph node [67]. Depletion of neutrophils during BCG vaccination abrogated the induction of Th1-specific responses and prohibited the reduction of bacterial load observed in vaccinated animals [68]. After the onset of T cell response, the cross-talk between neutrophils and T lymphocytes is maintained by T cell secretion of cytokines and chemokines able to recruit neutrophils to the sites of infection, activate them, and support their survival (e.g., IL-17 [59]).

Several observations suggest a role for neutrophils in the early organization of granuloma [69]. Initial TB foci contain a mixture of neutrophils, macrophages, and lymphocytes [37, 38]. Neutrophils secrete a set of chemokines attracting monocytes and T lymphocytes, such as CXCL2, CXCL9/MIG, CXCL10/IP-10, CXCL11/I-TAC, CCL3, and CCL4. In neutrophil-depleted mice, the formation of granulomas was reduced in terms of their number, size, and density. Similar results were obtained in *Cxcr3*^−/−^ mice and mice treated with anti-MIG antibodies [70]. Lack of IL-17 hampered both neutrophil recruitment to the lungs and the generation of granulomas [71]. Neutrophils also played an important role in granuloma formation in a mouse model of hypersensitivity pneumonitis [72].

With the appearance of antigen-specific Th1 and Th17 cells, a mixture of immune cells present in the incipient granulomas becomes organized. The organized granulomas may evolve in different ways dominated by proliferative or exudative processes, resulting in the formation of structurally heterogeneous lesions, including tuberculoma, cavitary, fibro-calcified, necrotic, and other types of lesions. At this stage of disease, large-scale infiltration of neutrophils is associated with exudative processes, lesion necrosis, and bacillary growth [37, 38, 73, 74]. It has recently been shown that an enormous heterogeneity of tuberculosis lesions coexists within a single individual [73]. This raises a question on the underlying mechanisms, including those that regulate neutrophilic response within each particular granuloma.

Overall, at the early stage of *Mtb* infection, neutrophils may contribute to the protection by favoring the generation of effector T cells and participating in the formation of granulomas. Supporting data are generally uniform, but not numerous. Further analyses are needed to unravel mechanisms and the extent to which neutrophil response is implicated in these processes during the onset of *Mtb* infection. At later stages of disease, neutrophils become largely detrimental.

## 4. Neutrophils in Inflammation and Tissue Damage

### 4.1. General Properties

Bactericidal and proinflammatory molecules produced by neutrophils are needed to ensure host immune protection. However, the same factors can be detrimental and trigger tissue damage via multiple mechanisms. Particularly, ROI and chlorinated oxidants are directly cytotoxic and induce tissue necrosis. They also activate MMPs and inactivate the inhibitors of proteinases which multiplies the inflammatory process [19, 75, 76]. ROI are implicated in the generation of NETs. NET-associated molecules (i.e., MPO, elastase, histones, and proteases) destruct the connective tissue, cleave host proteins, degrade heparan sulfate proteoglycan, and are directly cytotoxic for endothelial and epithelial cells [25]. Proinflammatory cytokines and chemokines, such as IL-1*β*, TNF-*α*, CXCL8, CCL3, and CCL4, propagate the inflammation [25, 46, 47].

At a single-cell level, neutrophil secretion of proinflammatory cytokines is not high. However, it becomes prominent when the cells accumulate in high numbers, especially due to a positive feedback regulation of neutrophilic inflammation: most of the cytokines and chemokines released by neutrophils act as neutrophil activators and attractants. In steady-state conditions, neutrophils spontaneously die by apoptosis and are engulfed by macrophages during the efferocytosis process that dampens the inflammation. During infections, neutrophil apoptosis delays and activated neutrophils die by necrosis, which leads to a defective removal of dead cells and progressive tissue damage [77]. The main inhibitors of cytokine production by neutrophils are IL-10, IL-4, and IL-13 [19]. However, during TB, these factors are poorly produced, meaning that once initiated, neutrophilic inflammation would be difficult to terminate. Pathogen clearance is the main way to resolve neutrophilic inflammation, but in the case of TB, this is a slow process.

In summary, biological properties of neutrophils suggest their dual role during TB: (i) providing a mechanism for bacteria killing, participating in the generation of acquired immunity and immune cell cooperation, and (ii) inducing hyperinflammatory response and tissue damage. In line with this dualism, data concerning neutrophil function during *Mtb* infection are highly contradictory.

### 4.2. Neutrophils in Inflammation and Pathology during Tuberculosis

While neutrophils have been associated with TB protection in some studies, most of the studies in the field are focused on their pathological role. In experimental setting, mice of TB-susceptible strains (i.e., I/St, DBA/2, and C3HeB/FeJ) developed extensive neutrophilic inflammation, which was not observed in more resistant mouse strains [27, 78–80]. Three studies examined immunological correlates of disease pathology using genetically heterogeneous populations of mice, that is, F2 mice generated by crossing TB-susceptible I/St and TB-resistant A/Sn mice, diversity outbred mice, and more recently highly diverse inbred mouse strains, consisting of the founder and recombinant strain progeny. In all three models, severe infection correlated strongly with the accumulation of neutrophil-like cells in the lungs [10, 81, 82].

In humans, active TB and disease severity have also been associated with neutrophilic response. In the study by Sutherland and coauthors, an increase in granulocytes and high granulocyte/lymphocyte ratio distinguished TB patients from tuberculin skin test-positive healthy contacts [83]. Berry and coauthors identified overexpression of IFN-inducible genes in purified blood neutrophils as the main transcript signature of active TB [84]. Within the group of TB patients, extensive neutrophilic response is a sign of TB severity and, specifically, has been associated with pulmonary destruction. Barry and coauthors showed that tuberculosis cavities contain more neutrophils and less lymphocytes compared to undestructive pulmonary infiltrates and radiologically unaffected lobes of the lungs [85]. In line with this, neutrophil-derived collagenase MMP-8 was upregulated in TB patients and caused matrix destruction in vitro and in respiratory samples of TB patients [86]. During pleural TB, the accumulation of neutrophils in pleural effusions was associated with significantly higher inflammatory serum markers and a more frequent detection of *Mtb* in pleural fluid and smear, thus linking neutrophils, intense inflammatory response, and a degree of pathogen excretion/load [87].

An association between neutrophil recruitment and overproduction of inflammatory cytokines and chemokines has been observed in many studies. In F2 hybrid mice and diverse outbred mice, an enhanced infiltration of the lung tissue with neutrophil-like cells coincided with the exuberant pulmonary expression of IL-1*β*, IL-6, CCL3, CCL4, MMP-8, and other factors [10, 81]. Mice susceptible to *Mtb* infection due to deletion of various genes developed both enhanced neutrophilic infiltration and overexpression of many inflammatory factors. Some examples are provided below.

IL-18 is involved in the generation of IFN-*γ*-producing CD4 and cytotoxic CD8 T cells. Mice deficient in IL-18 promptly succumbed to *Mtb* infection. Besides having decreased Th1 response, they exhibited neutrophilic (Gr-1^+^ cell) infiltration and enhanced protein and/or mRNA levels of IL-6, IL-17, CXCL1, CXCL2, CCL2, and CCL3 in the sera and the lung tissue [88].

CARD9 is an adaptor molecule that samples signals from pattern recognition receptors and mediates activation of innate immunity. After aerosol *Mtb* challenge, *Card*^−/−^ mice succumbed early to the infection with higher *Mtb* burden, accelerated granulocyte (MPO-expressing cell) recruitment, and higher abundance of the proinflammatory factors CCL2, CXCL1, and G-CSF [89].

Macrophage migration inhibitory factor (MIF) is an innate cytokine released by macrophages, lymphocytes, and pulmonary epithelial cells in response to microbial stimuli. *Mif*^−/−^ mice succumbed more quickly to *Mtb* infection with higher *Mtb* burden, increased pulmonary neutrophil accumulation, and increased production of CXCL2 and G-CSF (albeit a decreased production of TNF-*α*, IL-12, and IL-10) [90].

Bone marrow chimeric mice with IFN-*γ*-unresponsive lung epithelial and endothelial cells exhibited earlier mortality and higher bacterial burdens than control mice. The chimeric mice developed massive neutrophilic inflammation in the lungs accompanied by overproduction of CCL3, CXCL2, CXCL5, IL-1*β*, MMP-9, and other inflammatory factors [91].

C-type lectin receptor Mincle is involved in neutrophil migration driven by TDM. In *Mincle*^−/−^ mice challenged with TDM, neutrophils did not accumulate in the lungs and the mice had decreased mRNA levels of IL-6, TNF-*α*, and CXCL2 in the lungs [45].

In *Mtb*-infected *Cxcl5*^−/−^ mice, enhanced survival was accompanied by impaired neutrophil recruitment and decreased levels of CXCL1, CXCL2, CCL2, CCL3, CCL4, and CXCL10 in the bronchoalveolar lavage fluid [92].

There are multiple pathways whereby neutrophils and excessive inflammation may induce tissue pathology. Among them, the breakdown of extracellular matrix seems to be the main that leads to pulmonary destruction [93]. Neutrophil-derived MMP-8 is one of the main players in this process [86, 94].

Overall, there is an undeniable association between neutrophilic infiltration and the exuberant production of proinflammatory cytokines/chemokines at advanced TB stages. The underlying mechanisms are bidirectional and form a positive self-amplifying feedback loop: neutrophils transcribe a wide range of proinflammatory proteins attracting immune cells at the site of infection and activating them [19, 61, 95]; immune, endothelial, and epithelial cells secrete proinflammatory cytokines and chemokines that influence neutrophil differentiation, mobilization, and recruitment. Particularly, CXCL1, CXCL2, CXCL5, TNF-*α*, and G/GM-CSF stimulate neutrophil migration; TNF-*α* and IFN-*γ* activate neutrophils for cytokine/chemokine production; and IL-1*β*, G/GM-CSF, and IL-3 prolong neutrophil survival and are involved in granulopoiesis [19, 20, 61, 96]. Both the level of inflammation and intrinsic capacity of neutrophils to migrate to inflammatory stimuli were suggested as factors determining TB pathology [13, 78].

Of note, neutrophilic infiltration and exacerbated inflammation accompany severe disease in hosts with diverse genetic backgrounds and/or different gene mutations. This means that both processes are rather a result than an initial cause of disease progression. This and a positive feedback loop existing between both processes suggest that independently on the initial (genetic) factor(s) causing TB susceptibility, it might be possible to ameliorate the disease by interrupting neutrophil response. With this regard, in many studies, depletion of neutrophils at the advanced disease stage abrogated the inflammation and reversed *Mtb*-susceptible phenotype, posing neutrophils as the major mediators of dysfunctional responses during TB [97]. However, the results are, again, conflicting (discussed below).

To summarize, neutrophilic infiltration and exuberant inflammation represent characteristic features of severe TB pointing to the commonality of the immunopathological pathways operating at advanced stages of disease in genetically different hosts. This provides an opportunity to develop strategies for host-directed therapy during TB irrespective of host genetic background and mechanisms underlying disease susceptibility.

## 5. Neutrophils during TB: Do Disease Stage and Cell Quantities Play a Role?

Large discrepancy of neutrophil data may partly be explained by their differential roles in the protection against *Mtb* infection and during TB disease and/or at early and advanced disease stages. Indeed, in humans, the background levels of neutrophils and HNP-1–3 correlated with the protection against active TB, whereas the accumulation of neutrophils in TB patients was associated with disease progression and pulmonary destruction [33, 41, 42, 83, 85, 86]. In a mouse model of TB, neutrophils were shown to enter the lungs in two waves, peaking around days 3 (T cell-independent wave) and 23–56 (T cell-dependent wave) postinfection [15, 28]. It has been suggested that early-wave neutrophils have high potential for pathogen clearance, whereas at later stages, the cells rather contribute to pathology [12, 39].

To unravel neutrophil function during *Mtb* infection, many experimental studies used a mean of cell depletion with neutralizing anti-Gr-1 or anti-Ly-6G antibodies. The results are highly controversial. Most studies that depleted neutrophils in TB-resistant mice (e.g., B6 or BALB/c) before and/or very early following the infection (up to day 4) reported increased bacillary loads and worsened disease, suggesting a contribution of neutrophils to mycobacterial control [98, 99]. However, in the study by Seiler and coauthors, neutrophil depletion did not affect mycobacterial CFUs and mice survival, but only hampered granuloma formation [70]. Keller and coauthors reported that early neutrophil depletion did not affect the disease in resistant B6 mice and had beneficial effect on susceptible DBA/2 mice [78].

The depletion of neutrophils later following the infection (starting day 7 or later) mostly led to beneficial effects. However, most of the studies utilized KO mice developing severe inflammatory and neutrophilic responses following *Mtb* infection. For example, late neutrophil depletion reversed inflammatory reactions in the lungs, ameliorated the disease, and significantly prolonged the survival of *Card*^−/−^, *Ifnar*^−/−^, and *Atg5^fl/fl^-LysM-Cre* mice [89, 97, 100]. In the study by Schneider and coauthors, the administration of neutrophil-neutralizing antibodies on days 10 and 16 postchallenge slightly decreased *Mtb* burden in susceptible *Il18*^−/−^ mice, but did not affect B6 mice [88].

Lombard et al. [28] analyzed the effects of neutrophil depletion on B6 mice challenged with different doses of *Mtb* or BCG. The depletion decreased the load of *Mtb* but not that of BCG. Of note, at the time of depletion, mice had similar bacterial loads but different neutrophil counts in the lungs (higher in *Mtb*-challenged mice). Thus, the data link the effect of neutrophil depletion with their background quantities.

Overall, the overview of depletion experiments shows that only partly can the contradictory results be explained by the differential role of neutrophils at early and advanced disease stages. Rather, the effects seem to depend on host TB susceptibility and the extent of the background neutrophilic/inflammatory responses. This hypothesis, however, explains the effects of late neutrophil depletion. The first neutrophil wave is usually transient, not exuberant and not associated with overwhelmed inflammation. Thus, it is still unclear why early depletion of neutrophils benefits the host (in some studies), why the effect depends on host TB susceptibility, and what are mechanisms mediating pathological effects of neutrophils at the early infection stage. One recent hypothesis suggests that NETs provide a platform for extracellular *Mtb* growth and that extracellular bacilli are responsible for the transition from infection to active TB [37]. This hypothesis, however, implies the existence of intrinsic differences between neutrophils from TB-resistant and TB-susceptible mice (independent on genetic factors implicated in TB susceptibility), which raises further questions.

To summarize, in humans, neutrophil response has been associated with both protection against TB disease and pulmonary pathology during the disease, suggesting a differential cell role at different stages of *Mtb* infection. In mice, depletion experiments give contradictory results that depend primarily not only on the level of the background inflammation but also on other factors that are not fully understood.

## 6. Neutrophils during TB: Are They the Same Cells as in Steady-State Conditions?

In mouse studies, neutrophils are most often identified based on the expression of Gr-1 (expressed by granulocytes and monocytes) or Ly-6G (known to be exclusively expressed by granulocytes). However, it was previously shown that *Mtb* infection dramatically decreases the levels of Gr-1/Ly-6G expression [10]. This suggested changes in neutrophil population during *Mtb* infection. The subsequent examination of Gr-1-/Ly-6G-expressing cells confirmed the hypothesis and discovered a heterogeneity of neutrophilic population during *Mtb* infection. Neutrophil heterogeneity and qualitative changes that these cells undergo during *Mtb* infection are an emerging area of research, and not many studies have been published in the field so far. A brief summary of available data is presented below.

### 6.1. Myeloid-Derived Suppressor Cells

Examination of cells with low Gr-1/Ly-6G expression (Gr-1^dim^ cells) accumulating in the lungs of *Mtb*-infected mice showed that the cells belong to immature myeloid population [101–103]. In the study by Tsiganov and coauthors, the cells coexpressed neutrophilic (Gr-1, Ly-6G), monocytic (F4-80), and myeloid (CD11b) cell markers and had immaturity signs, such as increased expression of CD117, CD135, and unsegmented nuclei. Functional characteristic of Gr-1^dim^ cells showed that they inhibited T cell proliferation and IFN-*γ* secretion in an NO-dependent manner; that is, the cells were myeloid-derived suppressor cells (MDSC) [102]. In the study by Knaul and coauthors, MDSC released proinflammatory (IL-6, IL-1*α*) and anti-inflammatory (IL-10) cytokines and were able to phagocyte *Mtb*; their depletion ameliorated *Mtb*-induced disease, suggesting that the cells could provide a niche for pathogen survival and tailor immunity in TB [103]. Gr-1^dim^ cells were shown to accumulate in different TB-susceptible necrosis-prone mouse strains (i.e., I/St, *Nos2*^−/−^, *Rag*^−/−^, and C3HeB/FeJ) [101, 102]. Of note, their accumulation was accompanied by a dramatic drop in the numbers of neutrophils expressing typical Gr-1/Ly-6G^hi^ phenotype [102]. Thus, one of the outcomes of these studies is an indication that severe TB may be associated with a deficiency in true neutrophils instead of their excess.

The accumulation of MDSC is not a trait of only experimental *Mtb* infection; it was also reported in TB patients. In humans, MDSC are identified as HLA-DR^−/low^CD11b^+^CD33^+^ cells expressing CD14 or CD15/CD66b markers (monocytic and granulocytic MDSC, resp.). TB patients were shown to have higher frequencies of granulocytic MDSC compared to healthy controls. MDSC obtained from TB patients suppressed T cell response in an NO-dependent manner and were associated with a higher inflammatory response in coculture (i.e., higher IL-1, IL-6, IL-8, G-CSF, and GM-CSF) [104, 105]. Successful TB treatment reduced MDSC population, suggesting a role for MDSC during active TB disease [105]. Due to a limited number of studies, it is not yet clear whether MDSC contribute to TB pathology (e.g., by dampening T cell-mediated protection and/or exuberating inflammation) or represent a regulatory population aimed to limit excessive immune response.

### 6.2. TB-Associated Neutrophils (TBAN)

Changes to neutrophil population occurring during TB disease are not limited only to the generation of MDSC. Another feature of mouse TB is an alteration of “Gr-1/Ly-6G^hi^” population. It has been demonstrated that cells that in *Mtb*-infected mice fall into the “Gr-1/Ly-6G^hi^” gates (TBAN) differ from Gr-1/Ly-6G^hi^ neutrophils found in steady-state conditions (“steady-state neutrophils” (SSN)): TBAN had lower expression levels of Gr-1/Ly-6G, elevated expression of immaturity markers CD115 and CD135, and, in contrast to SSN, were able to inhibit T cell proliferation [102]. It was previously shown that the levels of Gr-1 and Ly-6G expression correlate with the degree of granulocytic differentiation and maturation [106, 107]. Thus, the data strongly indicate on a less mature state of TBAN compared to that of SSN. Whether these changes alter neutrophil capacity for mycobacterial control during TB is not yet clear.

### 6.3. Low-Density Neutrophils

Further heterogeneity of neutrophil-like populations during TB comes from changes in their density. Low-density granulocytes (LDG) have been previously described in several pathological conditions, such as systemic lupus erythematosus, rheumatoid arthritis, asthma, and HIV infection, where elevated LDG levels correlated with disease severity [108–111]. A characteristic feature of LDG is their remaining in the PBMC layer after density-gradient centrifugation along with the expression of the main neutrophil markers (i.e., CD15, CD66b, and CD11b) and lack of the expression of CD14. Several studies reported that the pattern of mRNA transcripts in LDG, that is, the expression of granule enzymes and bactericidal proteins, is characteristic of immature neutrophils. Functional analyses showed decreased phagocytic activity of LDG, their enhanced capacity to form NETs, and increased secretion of proinflammatory cytokines, suggesting cell implication in the inflammatory and tissue damaging processes and pathogenesis of diverse diseases [112].

The first study examining LDG during TB has recently been published by Deng and coauthors [113]. The authors reported the accumulation of LDG in patients with active TB and higher LDG levels in patients with more advanced disease compared to those with mild-to-moderate disease. Interestingly, LDG could be generated in vitro from normal-density granulocytes (NDG) cultured in the presence of *Mtb*, allowing the authors to suppose that LDG were not immature neutrophils but represented activated mature neutrophils that had degranulated. This contrasts several other studies that examined LDG during other diseases and considered them immature granulocytes. Overall, the origin, signals inducing LDG generation, and their precise role during TB are unclear. However, there is no doubt that the cells represent a subset of neutrophilic cells and contribute to neutrophil heterogeneity during TB disease.

### 6.4. Neutrophil Heterogeneity in Cancer

While the heterogeneity of neutrophils during TB only starts to be appreciated, neutrophil diversity in other diseases, particularly, in cancer, has been studied in more details. In cancer, the existence of multiple subsets and phenotypes of neutrophils has been demonstrated, including MDSC, type 1 (N1) and type 2 (N2) neutrophils, and hybrid tumor-associated neutrophils (TAN) [114–116]. Most of these subsets express neutrophil-specific markers (CD15/CD66b) but differ by other phenotypic and functional characteristics and exhibit differential roles during the disease. Particularly, N1 are proinflammatory and antitumorogenic. In contrast, N2 are immunosuppressive and protumorogenic. “Hybrid” TAN exhibit characteristics of both neutrophils and antigen-presenting cells, originate from mature neutrophils in tumor microenvironment, and serve as antigen-presenting cells stimulating T cell response at the earliest stages of lung cancer [116, 117]. In contrast, granulocytic MDSC accumulate at the late cancer stages, are immature, and inhibit proliferation of activated autologous T cells and IFN-*γ* production [118]. Each of these subsets seems to be further heterogeneous with regard to the surface phenotype, nuclear morphology, and other characteristics. The relationships between differential neutrophil-like subsets are not fully understood. However, it was suggested that the existence of the subsets exhibiting sometimes opposing effects (e.g., inhibiting or stimulating T cells) may underlie the opposing functions of neutrophils in cancer.

In summary, neutrophil population is highly heterogeneous and composed of different subsets that differ by their maturity, phenotype, and functional properties. This heterogeneity documents alterations, which neutrophil population undergo during pathological conditions, including TB, and may underlie the controversy of the existing data on neutrophil function in tuberculosis.

## 7. Conclusions

Neutrophils are multifunctional cells and during TB play a dual role. Particularly, they participate in the generation of the acquired immunity and granuloma formation and may kill *Mtb*. At the same time, the cells can support *Mtb* growth; have been implicated in the transition from infection to active TB; and mediate tissue destruction, disease severity, and progression. It is largely assumed that protective activity of neutrophils is more pronounced at the early stage of disease, whereas at the advanced TB stages, neutrophils become detrimental. This “two-stage” concept raises several questions. Particularly, why neutrophil effects differ so profoundly depending on the stage of TB infection? If neutrophil can kill *Mtb* during the onset of the infection, why do they fail to do so at later infection stages? Based on the analysis of several recent studies, we suggest that TB disease dramatically alters neutrophil population, leading to the accumulation of heterogeneous subsets of immature and activated dysfunctional cells and a decline in true neutrophils. The origin of these cells, signals leading to their generation, and their precise role during TB are yet to be determined.

## References

[1] Cooper A. M. (2009). Cell-mediated immune responses in tuberculosis. *Annual Review of Immunology*.

[2] Flynn J. L., Chan J., Lin P. L. (2011). Macrophages and control of granulomatous inflammation in tuberculosis. *Mucosal Immunology*.

[3] Lebre M. C., Burwell T., Vieira P. L. (2005). Differential expression of inflammatory chemokines by Th1- and Th2-cell promoting dendritic cells: a role for different mature dendritic cell populations in attracting appropriate effector cells to peripheral sites of inflammation. *Immunology and Cell Biology*.

[4] Monin L., Khader S. A. (2014). Chemokines in tuberculosis: the good, the bad and the ugly. *Seminars in Immunology*.

[5] Caruso A. M., Serbina N., Klein E., Triebold K., Bloom B. R., Flynn J. L. (1999). Mice deficient in CD4 T cells have only transiently diminished levels of IFN-gamma, yet succumb to tuberculosis. *Journal of Immunology*.

[6] Cooper A. M., Dalton D. K., Stewart T. A., Griffin J. P., Russell D. G., Orme I. M. (1993). Disseminated tuberculosis in interferon gamma gene-disrupted mice. *The Journal of Experimental Medicine*.

[7] Flynn J. L. (1993). An essential role for interferon gamma in resistance to Mycobacterium tuberculosis infection. *The Journal of Experimental Medicine*.

[8] Gallant J. E., Ko A. H. (1996). Cavitary pulmonary lesions in patients infected with human immunodeficiency virus. *Clinical Infectious Diseases*.

[9] Bustamante J., Boisson-Dupuis S., Abel L., Casanova J.-L. (2014). Mendelian susceptibility to mycobacterial disease: genetic, immunological, and clinical features of inborn errors of IFN-γ immunity. *Seminars in Immunology*.

[10] Lyadova I. V., Tsiganov E. N., Kapina M. A. (2010). In mice, tuberculosis progression is associated with intensive inflammatory response and the accumulation of Gr-1 cells in the lungs. *PloS One*.

[11] Barber D. L., Mayer-Barber K. D., Feng C. G., Sharpe A. H., Sher A. (2011). CD4 T cells promote rather than control tuberculosis in the absence of PD-1-mediated inhibition. *Journal of Immunology*.

[12] Lyadova I. (2012). *Inflammation and Immunopathogenesis of Tuberculosis Progression, Understanding Tuberculosis - Analyzing the Origin of Mycobacterium Tuberculosis Pathogenicity*.

[13] Dorhoi A., Kaufmann S. H. E. (2015). Versatile myeloid cell subsets contribute to tuberculosis-associated inflammation. *European Journal of Immunology*.

[14] Sakai S., Kauffman K. D., Sallin M. A. (2016). CD4 T cell-derived IFN-γ plays a minimal role in control of pulmonary Mycobacterium tuberculosis infection and must be actively repressed by PD-1 to prevent lethal disease. *PLoS Pathogens*.

[15] Appelberg R., Silva M. T. (1989). T cell-dependent chronic neutrophilia during mycobacterial infections. *Clinical and Experimental Immunology*.

[16] Mayadas T. N., Cullere X., Lowell C. A. (2014). The multifaceted functions of neutrophils. *Annu rev Pathol Mech dis.*.

[17] Choi E. Y., Santoso S., Chavakis T. (2009). Mechanisms of neutrophil transendothelial migration. *Frontiers in Bioscience: A Journal and Virtual Library*.

[18] Futosi K., Fodor S., Mócsai A. (2013). Reprint of neutrophil cell surface receptors and their intracellular signal transduction pathways. *International Immunopharmacology*.

[19] Witko-Sarsat V., Rieu P., Descamps-Latscha B., Lesavre P., Halbwachs-Mecarelli L. (2000). Neutrophils: molecules, functions and pathophysiological aspects. *Laboratory Investigation*.

[20] Summers C., Rankin S. M., Condliffe A. M., Singh N., Peters A. M., Chilvers E. R. (2010). Neutrophil kinetics in health and disease. *Trends in Immunology*.

[21] Fu L. M. (2003). The potential of human neutrophil peptides in tuberculosis therapy. *The International Journal of Tuberculosis and Lung Disease*.

[22] Segal A. W. (2005). How neutrophils kill microbes. *Annual Review of Immunology*.

[23] Brinkmann V., Reichard U., Goosmann C. (2004). Neutrophil extracellular traps kill bacteria. *Science*.

[24] Almyroudis N. G., Grimm M. J., Davidson B. A., Röhm M., Urban C. F., Segal B. H. (2013). NETosis and NADPH oxidase: at the intersection of host defense, inflammation, and injury. *Frontiers in Immunology*.

[25] Porto B. N., Stein R. T. (2016). Neutrophil extracellular traps in pulmonary diseases: too much of a good thing?. *Frontiers in Immunology*.

[26] Kisich K. O., Higgins M., Diamond G., Heifets L. (2002). Tumor necrosis factor alpha stimulates killing of Mycobacterium tuberculosis by human neutrophils. *Infection and Immunity*.

[27] Eruslanov E. B., Lyadova I. V., Kondratieva T. K. (2005). Neutrophil responses to Mycobacterium tuberculosis infection in genetically susceptible and resistant mice. *Infection and Immunity*.

[28] Lombard R., Doz E., Carreras F., Subbian S. (2016). IL-17RA in non-hematopoietic cells controls CXCL-1 and 5 critical to recruit neutrophils to the lung of mycobacteria-infected mice during the adaptive immune response. *PloS One*.

[29] Ganbat D., Seehase S., Richter E. (2016). Mycobacteria infect different cell types in the human lung and cause species dependent cellular changes in infected cells. *BMC Pulmonary Medicine*.

[30] Denis M. (1991). Human neutrophils, activated with cytokines or not, do not kill virulent Mycobacterium tuberculosis. *The Journal of Infectious Diseases*.

[31] Jones G. S., Amirault H. J., Andersen B. R. (1990). Killing of Mycobacterium tuberculosis by neutrophils: a nonoxidative process. *The Journal of Infectious Diseases*.

[32] Sharma S., Verma I., Khuller G. K. (2000). Antibacterial activity of human neutrophil peptide-1 against Mycobacterium tuberculosis H37Rv: in vitro and ex vivo study. *The European Respiratory Journal*.

[33] Martineau A. R., Newton S. M., Wilkinson K. A. (2007). Neutrophil-mediated innate immune resistance to mycobacteria. *The Journal of Clinical Investigation*.

[34] Reyes-Ruvalcaba D., González-Cortés C., Rivero-Lezcano O. M. (2008). Human phagocytes lack the ability to kill Mycobacterium gordonae, a non-pathogenic mycobacteria. *Immunology Letters*.

[35] Corleis B., Korbel D., Wilson R., Bylund J., Chee R., Schaible U. E. (2012). Escape of Mycobacterium tuberculosis from oxidative killing by neutrophils. *Cellular Microbiology*.

[36] Ramos-Kichik V., Mondragón-Flores R., Mondragón-Castelán M. (2009). Neutrophil extracellular traps are induced by Mycobacterium tuberculosis. *Tuberculosis*.

[37] Cardona P.-J. (2016). The progress of therapeutic vaccination with regard to tuberculosis. *Frontiers in Microbiology*.

[38] Ehlers S., Schaible U. E. (2012). The granuloma in tuberculosis: dynamics of a host-pathogen collusion. *Frontiers in Immunology*.

[39] Lowe D. M., Redford P. S., Wilkinson R. J., O’Garra A., Martineau A. R. (2012). Neutrophils in tuberculosis: friend or foe?. *Trends in Immunology*.

[40] Tan B. H., Meinken C., Bastian M. (2006). Macrophages acquire neutrophil granules for antimicrobial activity against intracellular pathogens. *Journal of Immunology*.

[41] Eum S.-Y., Kong J.-H., Hong M.-S. (2010). Neutrophils are the predominant infected phagocytic cells in the airways of patients with active pulmonary TB. *Chest*.

[42] Zhu L.-M., Liu C.-H., Chen P. (2011). Multidrug-resistant tuberculosis is associated with low plasma concentrations of human neutrophil peptides 1-3. *The International Journal of Tuberculosis and Lung Disease*.

[43] Sugawara I., Udagawa T., Yamada H. (2004). Rat neutrophils prevent the development of tuberculosis. *Infection and Immunity*.

[44] Yang C.-T., Cambier C. J., Davis J. M., Hall C. J., Crosier P. S., Ramakrishnan L. (2012). Neutrophils exert protection in the early tuberculous granuloma by oxidative killing of mycobacteria phagocytosed from infected macrophages. *Cell Host & Microbe*.

[45] Lee W.-B., Kang J.-S., Yan J.-J., Deretic V. (2012). Neutrophils promote mycobacterial trehalose dimycolate-induced lung inflammation via the Mincle pathway. *PLoS Pathogens*.

[46] Cassatella M. A. (1995). The production of cytokines by polymorphonuclear neutrophils. *Immunology Today*.

[47] Scapini P., Lapinet-Vera J. A., Gasperini S., Calzetti F., Bazzoni F., Cassatella M. A. (2000). The neutrophil as a cellular source of chemokines. *Immunological Reviews*.

[48] Petrofsky M., Bermudez L. E. (1999). Neutrophils from Mycobacterium avium-infected mice produce TNF-alpha, IL-12, and IL-1 beta and have a putative role in early host response. *Clinical Immunology*.

[49] Riedel D. D., Kaufmann S. H. (1997). Chemokine secretion by human polymorphonuclear granulocytes after stimulation with Mycobacterium tuberculosis and lipoarabinomannan. *Infection and Immunity*.

[50] Matzer S. P., Baumann T., Lukacs N. W., Röllinghoff M., Beuscher H. U. (2001). Constitutive expression of macrophage-inflammatory protein 2 (MIP-2) mRNA in bone marrow gives rise to peripheral neutrophils with preformed MIP-2 protein. *Journal of Immunology*.

[51] Sawant K. V., McMurray D. N. (2007). Guinea pig neutrophils infected with Mycobacterium tuberculosis produce cytokines which activate alveolar macrophages in noncontact cultures. *Infection and Immunity*.

[52] Hilda J. N., Das S. D. (2016). TLR stimulation of human neutrophils lead to increased release of MCP-1, MIP-1α, IL-1β, IL-8 and TNF during tuberculosis. *Human Immunology*.

[53] Parks W. C., Wilson C. L., López-Boado Y. S. (2004). Matrix metalloproteinases as modulators of inflammation and innate immunity. *Nature Reviews. Immunology*.

[54] van Gisbergen K. P. J. M., Sanchez-Hernandez M., Geijtenbeek T. B. H., van Kooyk Y. (2005). Neutrophils mediate immune modulation of dendritic cells through glycosylation-dependent interactions between Mac-1 and DC-SIGN. *The Journal of Experimental Medicine*.

[55] Alfaro C., Suarez N., Oñate C. (2011). Dendritic cells take up and present antigens from viable and apoptotic polymorphonuclear leukocytes. *PloS One*.

[56] Beauvillain C., Delneste Y., Scotet M. (2007). Neutrophils efficiently cross-prime naive T cells in vivo. *Blood*.

[57] Abi Abdallah D. S., Egan C. E., Butcher B. A., Denkers E. Y. (2011). Mouse neutrophils are professional antigen-presenting cells programmed to instruct Th1 and Th17 T-cell differentiation. *International Immunology*.

[58] Duffy D., Perrin H., Abadie V. (2012). Neutrophils transport antigen from the dermis to the bone marrow, initiating a source of memory CD8+ T cells. *Immunity*.

[59] Kalyan S., Kabelitz D. (2014). When neutrophils meet T cells: beginnings of a tumultuous relationship with underappreciated potential. *European Journal of Immunology*.

[60] Tillack K., Breiden P., Martin R., Sospedra M. (2012). T lymphocyte priming by neutrophil extracellular traps links innate and adaptive immune responses. *Journal of Immunology*.

[61] Silva M. T. (2010). When two is better than one: macrophages and neutrophils work in concert in innate immunity as complementary and cooperative partners of a myeloid phagocyte system. *Journal of Leukocyte Biology*.

[62] Bratton D. L., Henson P. M. (2011). Neutrophil clearance: when the party is over, clean-up begins. *Trends in Immunology*.

[63] Greenlee-Wacker M. C. (2016). Clearance of apoptotic neutrophils and resolution of inflammation. *Immunological Reviews*.

[64] Filardy A. A., Pires D. R., Nunes M. P. (2010). Proinflammatory clearance of apoptotic neutrophils induces an IL-12(low)IL-10(high) regulatory phenotype in macrophages. *Journal of Immunology*.

[65] Mantovani A., Cassatella M. A., Costantini C., Jaillon S. (2011). Neutrophils in the activation and regulation of innate and adaptive immunity. *Nature Reviews. Immunology*.

[66] Blomgran R., Ernst J. D. (2011). Lung neutrophils facilitate activation of naive antigen-specific CD4+ T cells during Mycobacterium tuberculosis infection. *Journal of Immunology*.

[67] Abadie V., Badell E., Douillard P. (2005). Neutrophils rapidly migrate via lymphatics after Mycobacterium bovis BCG intradermal vaccination and shuttle live bacilli to the draining lymph nodes. *Blood*.

[68] Trentini M. M., de Oliveira F. M., Kipnis A., Junqueira-Kipnis A. P. (2016). The role of neutrophils in the induction of specific Th1 and Th17 during vaccination against tuberculosis. *Frontiers in Microbiology*.

[69] Silva Miranda M., Breiman A., Allain S., Deknuydt F., Altare F. (2012). The tuberculous granuloma: an unsuccessful host defence mechanism providing a safety shelter for the bacteria?. *Clinical & Developmental Immunology*.

[70] Seiler P., Aichele P., Bandermann S. (2003). Early granuloma formation after aerosol Mycobacterium tuberculosis infection is regulated by neutrophils via CXCR3-signaling chemokines. *European Journal of Immunology*.

[71] Umemura M., Yahagi A., Hamada S. (2007). IL-17-mediated regulation of innate and acquired immune response against pulmonary Mycobacterium bovis bacille Calmette-Guerin infection. *Journal of Immunology*.

[72] Ishizuka M., Miyazaki Y., Masuo M. (2015). Interleukin-17A and neutrophils in a murine model of bird-related hypersensitivity pneumonitis. *PloS One*.

[73] Barry C. E., Boshoff H. I., Dartois V. (2009). The spectrum of latent tuberculosis: rethinking the biology and intervention strategies. *Nature Reviews. Microbiology*.

[74] Lin P. L., Flynn J. L. (2010). Understanding latent tuberculosis: a moving target. *Journal of Immunology*.

[75] Weiss S. J. (1989). Tissue destruction by neutrophils. *The New England Journal of Medicine*.

[76] Parker H., Winterbourn C. C. (2013). Reactive oxidants and myeloperoxidase and their involvement in neutrophil extracellular traps. *Frontiers in Immunology*.

[77] McCracken J. M., Allen L.-A. H. (2014). Regulation of human neutrophil apoptosis and lifespan in health and disease. *J Cell Death.*.

[78] Keller C., Hoffmann R., Lang R., Brandau S., Hermann C., Ehlers S. (2006). Genetically determined susceptibility to tuberculosis in mice causally involves accelerated and enhanced recruitment of granulocytes. *Infection and Immunity*.

[79] Driver E. R., Ryan G. J., Hoff D. R. (2012). Evaluation of a mouse model of necrotic granuloma formation using C3HeB/FeJ mice for testing of drugs against Mycobacterium tuberculosis. *Antimicrobial Agents and Chemotherapy*.

[80] Marzo E., Vilaplana C., Tapia G., Diaz J., Garcia V., Cardona P.-J. (2014). Damaging role of neutrophilic infiltration in a mouse model of progressive tuberculosis. *Tuberculosis*.

[81] Niazi M. K. K., Dhulekar N., Schmidt D. (2015). Lung necrosis and neutrophils reflect common pathways of susceptibility to Mycobacterium tuberculosis in genetically diverse, immune-competent mice. *Disease Models & Mechanisms*.

[82] Smith C. M., Proulx M. K., Olive A. J. (2016). Tuberculosis susceptibility and vaccine protection are independently controlled by host genotype. *MBio*.

[83] Sutherland J. S., Jeffries D. J., Donkor S. (2009). High granulocyte/lymphocyte ratio and paucity of NKT cells defines TB disease in a TB-endemic setting. *Tuberculosis (Edinburgh, Scotland)*.

[84] Berry M. P. R., Graham C. M., McNab F. W. (2010). An interferon-inducible neutrophil-driven blood transcriptional signature in human tuberculosis. *Nature*.

[85] Barry S., Breen R., Lipman M., Johnson M., Janossy G. (2009). Impaired antigen-specific CD4+ T lymphocyte responses in cavitary tuberculosis. *Tuberculosis*.

[86] Ong C. W. M., Elkington P. T., Brilha S., Sassetti C. M. (2015). Neutrophil-derived MMP-8 drives AMPK-dependent matrix destruction in human pulmonary tuberculosis. *PLoS Pathogens*.

[87] Choi H., Chon H. R., Kim K., Subbian S. (2016). Clinical and laboratory differences between lymphocyte- and neutrophil-predominant pleural tuberculosis. *PloS One*.

[88] Schneider B. E., Korbel D., Hagens K. (2010). A role for IL-18 in protective immunity against Mycobacterium tuberculosis. *European Journal of Immunology*.

[89] Dorhoi A., Desel C., Yeremeev V. (2010). The adaptor molecule CARD9 is essential for tuberculosis control. *The Journal of Experimental Medicine*.

[90] Das R., Koo M.-S., Kim B. H. (2013). Macrophage migration inhibitory factor (MIF) is a critical mediator of the innate immune response to Mycobacterium tuberculosis. *Proceedings of the National Academy of Sciences*.

[91] Desvignes L., Ernst J. D. (2009). Interferon-γ-responsive nonhematopoietic cells regulate the immune response to Mycobacterium tuberculosis. *Immunity*.

[92] Nouailles G., Dorhoi A., Koch M. (2014). CXCL5-secreting pulmonary epithelial cells drive destructive neutrophilic inflammation in tuberculosis. *The Journal of Clinical Investigation*.

[93] Al Shammari B., Shiomi T., Tezera L. (2015). The extracellular matrix regulates granuloma necrosis in tuberculosis. *The Journal of Infectious Diseases*.

[94] Teixeira L. R., Dias M. B., Sales R. K. B. (2016). Profile of metalloproteinases and their association with inflammatory markers in pleural effusions. *Lung*.

[95] Gopal R., Monin L., Torres D. (2013). S100A8/A9 proteins mediate neutrophilic inflammation and lung pathology during tuberculosis. *American Journal of Respiratory and Critical Care Medicine*.

[96] Ueda Y., Cain D. W., Kuraoka M., Kondo M., Kelsoe G. (2009). IL-1R type I-dependent hemopoietic stem cell proliferation is necessary for inflammatory granulopoiesis and reactive neutrophilia. *Journal of Immunology*.

[97] Kimmey J. M., Huynh J. P., Weiss L. A. (2015). Unique role for ATG5 in neutrophil-mediated immunopathology during M. tuberculosis infection. *Nature*.

[98] Pedrosa J., Saunders B. M., Appelberg R., Orme I. M., Silva M. T., Cooper A. M. (2000). Neutrophils play a protective nonphagocytic role in systemic Mycobacterium tuberculosis infection of mice. *Infection and Immunity*.

[99] Appelberg R., Castro A. G., Gomes S., Pedrosa J., Silva M. T. (1995). Susceptibility of beige mice to Mycobacterium avium: role of neutrophils. *Infection and Immunity*.

[100] Dorhoi A., Yeremeev V., Nouailles G. (2014). Type I IFN signaling triggers immunopathology in tuberculosis-susceptible mice by modulating lung phagocyte dynamics. *European Journal of Immunology*.

[101] Obregón -Henao A., Henao-Tamayo M., Orme I. M., Ordway D. J., Khader S. (2013). Gr1intCD11b+ myeloid-derived suppressor cells in Mycobacterium tuberculosis infection. *PloS One*.

[102] Tsiganov E. N., Verbina E. M., Radaeva T. V. (2014). Gr-1dimCD11b+ immature myeloid-derived suppressor cells but not neutrophils are markers of lethal tuberculosis infection in mice. *Journal of Immunology*.

[103] Knaul J. K., Jörg S., Oberbeck-Mueller D. (2014). Lung-residing myeloid-derived suppressors display dual functionality in murine pulmonary tuberculosis. *American Journal of Respiratory and Critical Care Medicine*.

[104] du Plessis N., Loebenberg L., Kriel M. (2013). Increased frequency of myeloid-derived suppressor cells during active tuberculosis and after recent Mycobacterium tuberculosis infection suppresses T-cell function. *American Journal of Respiratory and Critical Care Medicine*.

[105] El Daker S., Sacchi A., Tempestilli M., Hoshino Y. (2015). Granulocytic myeloid derived suppressor cells expansion during active pulmonary tuberculosis is associated with high nitric oxide plasma level. *PloS One*.

[106] Hestdal K., Ruscetti F. W., Ihle J. N. (1991). Characterization and regulation of RB6-8C5 antigen expression on murine bone marrow cells. *Journal of Immunology*.

[107] Satake S., Hirai H., Hayashi Y. (2012). C/EBPβ is involved in the amplification of early granulocyte precursors during candidemia-induced “emergency” granulopoiesis. *Journal of Immunology*.

[108] Wright H. L., Makki F. A., Moots R. J., Edwards S. W. (2017). Low-density granulocytes: functionally distinct, immature neutrophils in rheumatoid arthritis with altered properties and defective TNF signalling. *Journal of Leukocyte Biology*.

[109] Denny M. F., Yalavarthi S., Zhao W. (2010). A distinct subset of proinflammatory neutrophils isolated from patients with systemic lupus erythematosus induces vascular damage and synthesizes type I IFNs. *Journal of Immunology*.

[110] Fu J., Tobin M. C., Thomas L. L. (2014). Neutrophil-like low-density granulocytes are elevated in patients with moderate to severe persistent asthma. *Annals of Allergy, Asthma & Immunology*.

[111] Cloke T., Munder M., Taylor G., Müller I., Kropf P. (2012). Characterization of a novel population of low-density granulocytes associated with disease severity in HIV-1 infection. *PloS One*.

[112] Carmona-Rivera C., Kaplan M. J. (2013). Low-density granulocytes: a distinct class of neutrophils in systemic autoimmunity. *Seminars in Immunopathology*.

[113] Deng Y., Ye J., Luo Q., Scriba T. J. (2016). Low-density granulocytes are elevated in mycobacterial infection and associated with the severity of tuberculosis. *PloS One*.

[114] Deniset J. F., Kubes P. (2016). Recent advances in understanding neutrophils. *F1000Research*.

[115] Fridlender Z. G., Sun J., Kim S. (2009). Polarization of tumor-associated neutrophil phenotype by TGF-β: “N1” versus “N2” TAN. *Cancer Cell*.

[116] Singhal S., Bhojnagarwala P. S., O’Brien S. (2016). Origin and role of a subset of tumor-associated neutrophils with antigen-presenting cell features in early-stage human lung cancer. *Cancer Cell*.

[117] Eruslanov E. B., Bhojnagarwala P. S., Quatromoni J. G. (2014). Tumor-associated neutrophils stimulate T cell responses in early-stage human lung cancer. *The Journal of Clinical Investigation*.

[118] Wu P., Wu D., Ni C. (2014). γδT17 cells promote the accumulation and expansion of myeloid-derived suppressor cells in human colorectal cancer. *Immunity*.

